# The Self-Administered Version of the Structured Interview for Disorders of Extreme Stress (SIDES-SR) in a Clinical and Non-Clinical Portuguese Sample

**DOI:** 10.3390/bs12120468

**Published:** 2022-11-22

**Authors:** Rute Pires, Ana Sousa Ferreira, Marco Paulino, João Gama Marques, Joana Henriques-Calado, Bruno Gonçalves

**Affiliations:** 1 CICPSI, Faculdade de Psicologia, Universidade de Lisboa, Alameda da Universidade, 1649-013 Lisboa, Portugal; 2 Faculdade de Psicologia, Universidade de Lisboa, 1649-013 Lisboa, Portugal; 3 Business Research Unit, Instituto Universitário de Lisboa (ISCTE-IUL), Av. das Forças Armadas, 1649-026 Lisboa, Portugal; 4 Faculdade de Medicina, Clínica Universitária de Psiquiatria e Psicologia Médica, Universidade de Lisboa, Avenida Professor Egas Moniz, 1649-028 Lisboa, Portugal; 5 Consulta de Esquizofrenia Resistente, Hospital Júlio de Matos, Centro Hospitalar Psiquiátrico de Lisboa, Av. do Brasil, 53, 1749-002 Lisboa, Portugal

**Keywords:** DESNOS, Portuguese version of the SIDES-SR, psychometric properties

## Abstract

Disorders of Extreme Stress Not Otherwise Specified (DESNOS) refers to a constellation of symptoms deriving from chronic interpersonal trauma, inflicted by caregivers or in the context of intimate relationships. The Structured Interview for Disorders of Extreme Stress—Self-Report (SIDES-SR) was designed for the assessment of DESNOS. This study aimed to validate the Portuguese version of the SIDES-SR in the community (*N* = 814; *M*_age_ = 40.09, *SD* = 14.25, 39.2% male, 60.8% female) and clinical (*N* = 310; *M*_age_ = 42.49, *SD* = 12.47, 57.7% male, 42.3% female) samples. It had three objectives: (1) to validate the SIDES-SR rationally derived domains in the community sample; (2) to characterise the reliability of the SIDES-SR scales in both samples; and (3) to explore mean differences in the SIDES-SR results in both samples. The Portuguese SIDES-SR confirmed the six clinical domains of DESNOS and demonstrated acceptable internal consistency levels, similar to those obtained in prior research. Highly significant differences and large and very large effect sizes between the community and clinical samples were found for all the SIDES-SR domains. DESNOS symptomatology was shown to be more frequent in females and the clinical sample reported a higher frequency of traumatic events in life, specifically interpersonal trauma. The results support the relevance of the SIDES-SR for clinical practice in the assessment of the DESNOS diagnosis.

## 1. Introduction

Disorders of extreme stress not otherwise specified (DESNOS) [1], also known as complex PTSD [2], developmental trauma disorder [3], or complex developmental trauma [4] refer to a constellation of symptoms and altered beliefs that emerge as a consequence of prolonged, repeated, adverse interpersonal experiences (e.g., child abuse, domestic violence). These experiences, or type II traumas [5], may compromise the individual’s self-development, particularly when they occur during developmentally vulnerable periods, such as early childhood or adolescence, and when they are inflicted by caregivers or in the context of other intimate relationships [6,7,8]. Indeed, a large proportion of adult psychiatric patients (40–70%) report a history of prolonged abuse, particularly in childhood [2,8].

There is still controversy over whether DESNOS and post-traumatic stress disorder (PTSD) [9] are distinct diagnoses, or whether the former is a complication of the latter [10]. Considering that most adults with DESNOS also meet PTSD criteria [11], the latest edition of the Diagnostic and Statistical Manual of Mental Disorders (DSM-5) [9] has included DESNOS psychopathology outcomes as specific features of PTSD. However, findings from clinical and research studies suggest that a PTSD diagnosis fails to adequately capture DESNOS’ constellation of symptoms [1,2,3,4,5,8,12,13,14,15,16,17,18,19,20,21] as PTSD emerges as a consequence of single-event traumas, or type I traumas [5], not directly attributed to another person’s abuse or neglect (e.g., natural disasters, vehicle accidents). PTSD psychopathology outcomes include intrusive memories, avoidance of the stimuli associated with the traumatic event, emotional numbing, arousal and emotional reactivity associated with the traumatic event [22]. On the other hand, DESNOS symptomatology is complex and extremely distressful for the individual, who usually receives several other diagnoses in comorbidity (e.g., depressive disorders, panic disorders). Victims of trauma who meet the criteria for DESNOS have problems regulating emotional experiences and display compulsive behaviours (i.e., eating, sexual activity, self-injury) as a way of dealing with overwhelming emotions [8]; tend to cope with adverse experiences by detaching them from consciousness (i.e., dissociation), which causes attention or consciousness disturbances [8]; have several somatic complaints (i.e., digestive, cardiopulmonary and urogenital symptoms) that challenge conventional medicine, partly due to the fact that given their extreme difficulty in verbalizing and coping with these traumatic experiences, the body becomes the “stage” upon which the unrecognised psychological suffering can be displayed [8,23]. Moreover, individuals with DESNOS have distorted beliefs regarding themselves, others and the meaning and purpose of life. In addition to questioning the meaning of life, they have a negative image of themselves and tend to repeat and renew past abusive relationships, not recognizing subtle signs of disrespect that are likely to turn into abuse in their present relationships [8].

Research on DESNOS is still scarce while most research on trauma is related to PTSD. There is substantial evidence that females are more prone than males to developing PTSD after exposure to a traumatic event [24,25,26,27,28,29,30], although males have a higher risk of experiencing a traumatic event in life [31]. Perhaps this latter difference is related to the type of trauma experienced, as males have been found to have greater exposure to war situations, to work, leisure and car accidents, while females are more likely to be at risk of sexual trauma [32].

Given the relevance of the DESNOS diagnosis for clinical practice, Pelcovitz et al. [1] designed the Structured Interview for Disorders of Extreme Stress (SIDES), a rationally derived structured interview that assesses lifetime and the current presence of DESNOS, in addition to the current symptom severity (past month), associated with exposure to chronic developmental trauma. In its prevailing form, there are two versions of the instrument, namely the interview (SIDES-R) and the self-administered version (SIDES-SR) [8]. Both comprise 45 items that assess six domains: (1) alterations in the regulation of affect and impulses, (2) alterations in attention or consciousness, (3) alterations in self-perceptions, (4) alterations in relationships with others, (5) somatisation, and (6) alterations in systems of meaning. The SIDES-R has demonstrated adequate levels of inter-rater reliability (*k* = 0.81), internal consistency (0.76 to 0.90, for the subscales; 0.96, for the full scale), and evidence of criterion-related validity, given that SIDES-R scores have been found to be positively related to a younger age at the time of the first victimisation, and to a history of chronic developmental trauma, particularly interpersonal violence [3]. Subsequent research on the psychometric properties of the SIDES-R has provided incremental evidence of its convergent validity with several related measures (e.g., SCL-90-R, amongst others) [33]. However, the rationally derived six-factor structure of the SIDES-R has neither been supported in the West [22] nor in non-Western countries [34], calling into question the universality of the construct [4].

To our knowledge, there are even fewer studies on the self-administered form of the Structured Interview for Disorders of Extreme Stress (SIDES-SR) and therefore, the current study was conducted to address the psychometric properties (construct validity and reliability) of the Portuguese version of the SIDES-SR in clinical and non-clinical samples. The study had three objectives: (1) to test and validate the SIDES-SR rational model; (2) to examine the reliability of the Portuguese version of the SIDES-SR in non-clinical and clinical samples; (3) to validate the Portuguese version of the SIDES-SR, by comparing the psychiatric patients’ results in the SIDES-SR to those obtained in the community sample. Gender differences and the presence of interpersonal trauma inflicted by caregivers or in the context of intimate relationships were also addressed. 

The following outcomes were expected (a) the Portuguese version of the SIDES-SR would demonstrate a six-factor structure and internal consistency levels, similar to those obtained with the original test [1,8]; (b) the Portuguese SIDES-SR would demonstrate structure invariance between the community and clinical samples and would differentiate both samples, with the clinical sample presenting more severe DESNOS symptoms than the community sample [2,8]; (c) females would experience more severe DESNOS symptoms than males [24,25,26,27,28,29,30]; (d) victims of interpersonal trauma would present more severe DESNOS symptoms than those who had not experienced interpersonal trauma [11]. 

## 2. Method

### 2.1. Participants and Procedure

The community sample consisted of 814 adults predominantly from an urban area, recruited from the relatives and acquaintances of undergraduate students from a higher education institution. This student-recruited sampling method can be considered a variant of the snowball sampling technique. This method was adopted given that it is an easy and inexpensive means of obtaining a large sample [35]. 

The clinical sample was composed of 310 patients. At the time of data collection, the patients were having treatment at mental health units and the diagnoses were attributed by the psychiatrist responsible for each patient, according to the DSM-5 classification. Intellectual disability, schizophrenia, and major and mild neurocognitive disorders diagnoses were the exclusion criteria. 

The community and clinical samples’ data collection was approved by the Ethics Committee of the researchers’ affiliation institution and, in the case of the clinical sample, by the host institutions involved. Informed consent was presented in written form and signed by both researchers and participants at the moment of data collection. Participants were informed that their collaboration was voluntary, that they could give up at any time, that no identifying information would be asked, and that the data would be used in a scientific study. In order to characterise the sample, the participants answered a sociodemographic questionnaire that included four questions on the traumatic experience: if they had had a traumatic experience, how many, when and what kind of traumatic experience it had been. The content of the answers to this last question was analysed and transformed into a quantitative variable related to the presence of relational trauma vs. common trauma. Relational trauma was coded whenever a participant reported one or more adverse interpersonal experiences (i.e., abuse, neglect or domestic violence), inflicted by caregivers or in the context of other intimate relationships, occurring in childhood and/or adolescence. Common trauma was coded whenever a participant reported one or more traumatic experiences (i.e., natural disasters, car accidents, war, death and disease) not caused by the behaviour of significant others.

### 2.2. Measures

SIDES-SR: Self-Report Inventory of Disorders of Extreme Stress [8,36]. 

The SIDES-SR is a 45-item self-report measure which assesses lifetime and current presence of DESNOS, in addition to current symptom severity (past month). Lifetime presence of DESNOS is rated on a dichotomic scale (Yes/No) while current presence in the past month and symptom severity are rated on a 4-point Likert scale, ranging from 0 (none; not at all) to 3 (maximum severity). Scores of 2 or above suggest the clinical severity of the symptoms presented. The SIDES-SR characterises DESNOS’ six symptomatology clusters: (I) alterations in regulation of affect and impulses (19 items), (II) alterations in attention or consciousness (5 items), (III) alterations in self-perception (6 items), (IV) alterations in relationships with others (5 items), (V) somatisation (5 items), (VI) alterations in systems of meaning (5 items). The SIDES-SR has demonstrated acceptable to high levels of internal consistency (0.74 to 0.82) for all subscales, except for the somatisation subscale (0.68) [8]. 

### 2.3. Data Analysis

The statistical analysis was conducted with IBM SPSS Statistic (Version 25) and with R (Version 3.6.0). Descriptive statistics for the SIDES-SR scales were obtained and internal reliability was examined through Cronbach’s alphas in both the community and clinical samples. In order to test and validate the SIDES-SR structure, a confirmatory factor analysis (CFA) was performed on the community sample, using the R package lavaan (latent variable analysis; version 0.6–5) and the following model fit indices: chi-square/degree of freedom (χ^2^/df), goodness fit index (GFI), comparative fit index (CFI), Tucker–Lewis index (TLI), normed fit index (NFI), root mean square error of approximation (RMSEA) and standardised root mean square residual (RMSR). Good fit model criteria were: χ^2^/df < 2, GFI, CFI, TLI and NFI > 0.90, RMSEA < 0.08 and RMSR ≈ 0 [37,38]. CFA was also performed on the clinical sample, so that the SIDES-SR structure invariance between the community and clinical samples could be measured and the SIDES-SR results in both samples compared. Factor structure, loading invariance and intercepts invariance were tested to establish structure invariance. 

The Student’s *t*-test and Cohen’s *d* were used to compare age and SIDES-SR results in both samples. The chi-square homogeneity test and phi measure were used for the remaining demographic variables. The effect size Cohen’s *d* was considered small when: *d* ≤ 0.20, medium when: 0.20 < *d* ≤ 0.50, large when: 0.50 < *d* ≤ 1.0, and very large when *d* > 1.0 (Cohen, 1988) and phi, small when: phi ≤ 0.30, medium when: 0.30 < phi ≤ 0.50, large when: phi > 0.50 [39]. 

## 3. Results

The main characteristics of the community and clinical samples are presented in Table 1.

The effects of age and gender in the community and clinical samples, while statistically significant, are small, supporting gender comparisons (objective 3). Heterogeneity is observed regarding the other demographic variables, however, the impact of these variables on SIDES-SR results is, to the best of our knowledge, unknown.

Considering trauma exposure, in the community sample, 45.0% of the participants reported having experienced one or more traumatic experiences during their lifetime, and 11.8% reported interpersonal trauma inflicted by caregivers or in the context of other intimate relationships. In the clinical sample, 73.1% of the participants reported having experienced one or more traumatic events in their life. From this subsample, 28.1% reported having experienced interpersonal trauma inflicted by caregivers or in the context of other intimate relationships.

### 3.1. Objective 1

In order to test and validate the SIDES-SR rationally derived domains, a confirmatory factor analysis (CFA) was performed on the community sample. CFA was applied using the R software, a free software environment for statistical computing and graphics. This factor analysis was conducted with missing data handled by listwise deletion, since missing data are completely random according to Little’s test, and the items’ missing rate is less than 5% [40]. An exploratory data analysis of the SIDES-SR observed variables revealing major deviations from normality in their distributions and exhibiting skewed distributions [41]. The CFA analysis was conducted with the lavaan package [42] using the weighted least squares means and variances (WLSMV) estimation method that uses diagonally weighted least squares (DWLS) to estimate the model parameters. The estimation method was selected to consider the ordinal level of measurement of the 45 items and their non-normal distributions. The six-factor hypothesised model of the SIDES-SR demonstrated an adequate to excellent fit to the data (robust index values: χ^2^/df = 1.01; CFI = 0.992; TLI = 0.992; NFI = 0.924; RMSEA = 0.004; P(rmsea ≤ 0.05) = 1.00, CI90 [0.00; 0.012]), since the CFI and TLI values were above 0.95, the NFI value was above 0.90, and the RMSEA value below 0.08 [37,38], while the factor loadings of all the items except item 9 were above 0.40. Factor loadings for the alterations in the regulation of affect and impulses items varied between 0.82 and 0.26; factor loadings for the alterations in the attention or consciousness items varied between 0.82 and 0.58; factor loadings for the alterations in the self-perception items varied between 0.76 and 0.51; factor loadings for the alterations in the relationships with others items varied between 0.79 and 0.67; factor loadings for the somatisation items varied between 0.80 and 0.65, and finally, factor loadings for the alterations in the systems of meaning items varied between 0.80 and 0.45. 

Table 2 presents SIDES-SR items’ factor loadings. 

To ensure that a six-dimensional structure was the best one for the SIDES-SR, a unidimensional CFA was performed. This unidimensional model also revealed an adequate fit to the data (robust index values: χ^2^/df = 1.20, CFI = 0.988; TLI = 0.988; NFI = 0.644; RMSEA = 0.005; P(rmsea ≤ 0.05) = 1.00, CI90 [0.00; 0.012]); however, the model with the six latent factors fit the data significantly better than the model with only a single latent factor (chi-square diff: χ^2^(15) = 36.73, *p* < 0.001).

### 3.2. Objective 2

Table 3 presents the SIDES-SR scales’ means (*M*), standard deviations (*SD*) and Cronbach’s alphas of the SIDES-SR scales in the community and clinical samples. The Portuguese version of the SIDES-SR revealed alphas ranging from 0.67 to 0.90 for the community sample and alphas between 0.62 and 0.77 for the clinical sample. 

### 3.3. Objective 3

In order to measure the SIDES-SR structure invariance between the community and clinical samples, a multiple-group confirmatory factor analysis [44] was conducted with the lavaan package. The six-factor hypothesised model of the SIDES-SR demonstrated an adequate fit to the clinical sample data (robust index values: χ^2^/df = 1.06, CFI = 0.968, TLI = 0.966, NFI = 0.901, RMSEA = 0.011, P(rmsea ≤ 0.05) = 1.00, CI90 [0.003; 0.016]), since the CFI and TLI values were above 0.95, the NFI value was above 0.90, and the RMSEA value below 0.08 [37,38]. In the clinical sample, the factor loadings for the alterations in the regulation of affect and impulses items varied between 0.76 and 0.26; factor loadings for the alterations in attention or consciousness items varied between 0.74 and 0.53; factor loadings for the alterations in self-perception items varied between 0.84 and 0.34; factor loadings for the alterations in relationships with others items varied between 0.76 and 0.41; factor loadings for the somatisation items varied between 0.89 and 0.65, and finally, factor loadings for the alterations in systems of meaning items varied between 0.93 and 0.50. 

Table 4 presents the measurement structure invariance analysis. 

The comparison of models 1 and 2 revealed non-significant χ^2^ changes and minimal fit index changes, indicating loading invariance. Similarly, the comparison of models 2 and 3 showed intercepts invariance; hence, structure invariance was established.

Since SIDES-SR structure invariance between the community and clinical samples was established, the means were then compared. Table 5 presents mean differences between the SIDES-SR results of the community and clinical samples. According to Cohen’s [39] criteria for interpreting effect magnitude, and despite the samples’ different sizes, the clinical sample differed appreciably from the community sample with respect to DESNOS’ constellation of symptoms.

Considering the participants’ reference to trauma, it was possible to conclude that in the community sample, there were no gender differences in the SIDES-SR domains for participants with or without trauma. However, in the clinical sample, a different scenario occurred for participants with or without trauma. In the clinical sample with no reference to trauma, a highly significant difference was observed for the alterations in attention or consciousness scale, *t*(40.98) = −2.72; *p* = 0.005; *d* = −0.72, with higher scores for females. In the clinical sample for the participants with reference to trauma, significant differences were observed for all but two SIDES-SR scales (i.e., alterations in relationships with others, *t*(210) = −0.54; *p* = 0.297; *d* = −0.08 and somatisation, *t*(198) = −1.71; *p* = 0.051; *d* = −0.20). Females had higher scores than males on the alterations in the regulation of the affect and impulses scale, *t*(190) = −2.35; *p* = 0.010; *d* = −0.33, on the alterations in attention or consciousness scale, *t*(205) = −3.15; *p* = 0.001; *d* = −0.45, on the alterations in self-perception scale, *t*(205) = −1.99; *p* = 0.024; *d* = −0.28 and on the alterations in systems of meaning scale *t*(203) = −1.86; *p* = 0.032; *d* = −0.26.

Concerning the differences in SIDES-SR scales depending on the type of trauma experienced (i.e., chronic interpersonal trauma or single-event trauma/common trauma), in the community sample, victims of chronic interpersonal trauma obtained higher scores than victims of single-event trauma on the alterations in the regulation of affect and impulses scale, *t*(43.51) = −2.39; *p* = 0.011; *d* = −0.52 and on the Alterations in self-perception scale, *t*(43.07) = −2.39; *p* = 0.011; *d* = −0.31. As for the clinical sample, victims of chronic interpersonal trauma obtained higher scores than victims of single-event trauma on the alterations in the regulation of affect and impulses scale, *t*(185) = −2.17; *p* = 0.016; *d* = −0.36 and on the alterations in attention or consciousness scale, *t*(201) = −2.51; *p* = 0.007; *d* = −0.38. 

## 4. Discussion

The current study addressed the validation of the Portuguese version of the Structured Interview for Disorders of Extreme Stress, in its self-administered form (SIDES-SR). It focused on the construct validity (objectives 1 and 3) and reliability (objective 2) of the Portuguese version of the test. 

Given that the six-factor structure of the SIDES-R has not been supported in prior research [22,34], factor analysis techniques were used to test the SIDES-SR model in the Portuguese community sample and revealed an adequate to excellent model fit (robust index values: χ^2^/df = 1.01; CFI = 0.992; TLI = 0.992; NFI = 0.924; RMSEA = 0.004; P(rmsea ≤ 0.05) = 1.00, CI90 [0.00; 0.012]), since the CFI and TLI values were above 0.95, the NFI value was above 0.90, and the RMSEA value below 0.08, while the factor loadings of all the items except item 9 were above 0.40. Therefore, our data provide empirical evidence for the SIDES-SR’s six clinical domains, thus contributing to the measure of construct validity. Moreover, having proven that the SIDES-SR has structure invariance between the community and clinical samples (results indicate the same factor structure, loading invariance and intercepts invariance, thus, structure invariance), the comparison of means highly differentiated both samples, with the clinical sample reporting, as expected, more severe symptomatology. Large to very large effect sizes suggest that, despite the *n* of the samples, the Portuguese version of the SIDES-SR adequately captures the severity of DESNOS symptomatology. 

In the clinical sample, females who reported the occurrence of trauma had significantly higher DESNOS symptomatology than males, in all but two SIDES-SR domains. Although prior research has corroborated females’ proneness to developing PTSD following exposure to a traumatic event [24,25,26,27,28,29,30], to our knowledge, research on gender differences in DESNOS symptomatology is scarce. The current findings confirm that, similar to PTSD, DESNOS symptomatology is also more frequent in females than in males. Considering the frequency and type of trauma experienced (i.e., chronic interpersonal trauma or single-event trauma), not only did the clinical sample report a higher frequency of one or more traumatic events in life than the community sample, it also reported more interpersonal trauma inflicted by caregivers or in the context of other intimate relationships. Chronic interpersonal trauma, occurring in the early stages of life, appears to be related to significantly higher scores on the alterations in the regulation of the affect and impulses scale, in both samples. 

As for the reliability of the Portuguese SIDES-SR, internal consistency indices ranged between 0.67 and 0.90 for the community sample, and between 0.62 and 0.77 for the clinical sample. Apart from the alterations in the regulation of the affect and impulses domain, the Portuguese version of the SIDES-SR revealed lower alphas than those obtained with the SIDES-R (alphas between 0.76 and 0.90) [1], but similar to data regarding the SIDES-SR [8], which also pointed to lower alphas (alphas between 0.68 and 0.82) than those obtained with the SIDES-R. In the same vein, Scoboria et al. [22] explored the factor structure of the SIDES-R and retained 20 items and five factors whose internal consistency was, for all but one factor, below 0.70, ranging from 0.67 to 0.71. In short, the Portuguese results regarding the internal consistency of the self-administered version of the SIDES’ domains were lower than those obtained with the original interview [1], but akin to other research findings with the self-administered version of the test [8] and with the interview [22]. These Cronbach’s alpha values may be a consequence of the complexity and heterogeneity of the DESNOS phenomenon. DESNOS’ scales, which comprise both clinical disorders (e.g., somatisation, dissociation and affective dysregulation) and pervasive patterns of inner experience and behaviour (e.g., long-lasting alterations in self-perception, relationships and systems of meaning), were constructed to accommodate the multidimensional nature of the DESNOS phenomenon. The scales’ lower internal consistency reliability may reflect the inherent breadth of the construct domains measured by each scale. Although the SIDES-SR scales are not highly unidimensional or homogeneous, they have, nonetheless acceptable reliabilities. It should be noted, however, that in our study, the alphas were lower in the clinical sample than in the community sample, perhaps due to the heterogeneity of the diagnoses in the clinical sample. Future research with more homogeneous clinical samples is necessary to clarify these alphas which may be seen as acceptable for exploratory studies [43], but are lower than those obtained in the community sample.

In a nutshell, and considering objectives 1, 2 and 3, the Portuguese data provided sufficient validity evidence of the test’s six-factor structure. The CFA showed that the six-factor SIDES-SR model had an adequate to excellent fit to the data, both in the community and clinical samples. Moreover, this study’s results show that the Portuguese SIDES-SR scales have acceptable reliabilities and can distinguish between clinical and non-clinical samples, with the clinical sample revealing significantly higher results in the SIDES-SR. In comparison to males, females from the clinical sample who reported trauma also obtained higher results on all but two of the SIDES-SR scales, thus suggesting more DESNOS symptomatology in females than in males. Finally, although the community sample reported less trauma and less relational trauma than the clinical sample, in both samples, victims of chronic interpersonal trauma obtained higher scores in some of the SIDES-SR scales, namely in the alterations in the regulation of affect and impulses, which is also, in both samples, the most reliable scale (0.90 in the community sample and 0.77 in the clinical sample).

This study has several limitations that should be considered when interpreting its findings. The composition of the clinical sample, in which the diagnosis of substance use is overrepresented, is one of its limitations. In future research, this sample will be expanded in order to balance the diagnoses. Another limitation of the current study is related to the potential hierarchical structure of the community data. Indeed, community-dwelling participants were recruited from the relatives and acquaintances of students, and this convenience sampling strategy may result in the presence of clusters in the sample (e.g., families). The fact that our community sample replicated the theoretically proposed six-factor structure contributed to the decision to not re-analyse the data relying on multilevel modelling techniques. Given the retrospective nature of trauma assessment, the fact that this study was based exclusively on self-rating instruments, in which response bias or socially desirable responses were not controlled, is another limitation. Moreover, the presence and frequency of traumatic experiences during a lifetime, and their interpersonal nature, were assessed through ad hoc questions; thus, they may also have led to distortions in recall. On a final note, evidence of convergent and discriminant validity with a PTSD measure was not addressed in this study, although it is one of the main anticipated future developments. The present findings do, however, suggest that the Portuguese version of the SIDES-SR has some clinical utility and may be useful in the assessment of DESNOS symptomatology. Therefore, in the future, further research will be conducted in order to replicate previous results and as mentioned above, to validate the SIDES-SR in relation to other trauma exposure measures. 

## Figures and Tables

**Table 1:** Community and clinical samples’ demographic characteristics.

		Community Sample (*N* = 814)	Clinical Sample (*N* = 310)	Statistics	*p*	Effect Meas.
Age (years)	*M*	40.09	42.49	−2.73	0.004	0.17
	*SD*	14.25	12.47			
Sex	Male	39.2%	57.7%	31.32	0.000	0.17
	Female	60.8%	42.3%			
Marital status	Unmarried	36.9%	53.7%	142.48	0.000	0.36
	Married or cohabiting	52.5%	16.5%			
	Widow	2.1%	2.3%			
	Separated/ Divorced	8.5%	27.5%			
Employment status	Employed	87.4%	34.9%	369.18	0.000	0.58
	Retired	5.2%	8.6%			
	Unemployed	6.0%	55.9%			
	Housewife	1.4%	0.7%			
Schooling	<9 years	7.5%	25.0%	100.07	0.000	0.30
	9 years	15.1%	21.7%			
	12 years	28.7%	31.1%			
	Univ. degree	48.8%	22.3%			
Diagnosis (Clinical sample)	Substance-related and addictive disorders	--	35.5%			
	Personality disorders		23.9%			
	Depressive disorders		18.7%			
	Feeding and eating disorders		8.7%			
	Bipolar and related disorders		5.8%			
	Anxiety disorders		5.2%			
	Obsessive Compulsive Disorders		4.5%			
	Disruptive, impulse control and conduct disorders		1.8%			

*Note.* The effect size Cohen’s *d* was considered small when: *d* ≤ 0.20, medium when: 0.20 < *d* ≤ 0.50, large when: 0.50 < *d* ≤ 1.0, and very large when *d* > 1.0; the effect size phi, was considered small when: phi ≤ 0.30, medium when: 0.30 < phi ≤ 0.50, large when: phi > 0.50.

**Table 2:** SIDES-SR’ items factor loadings.

Factor	Items	Loadings	*SE*	*z*	*p*
Alterations in regulation of affect and impulses	1	0.69	0.04	15.74	<0.001
	2	0.78	0.05	16.11	<0.001
	3	0.70	0.06	12.82	<0.001
	4	0.67	0.05	12.45	<0.001
	5	0.82	0.07	12.09	<0.001
	6	0.71	0.06	12.02	<0.001
	7	0.50	0.07	7.04	<0.001
	8	0.75	0.16	4.81	<0.001
	9	0.26	0.05	5.39	<0.001
	10	0.75	0.06	12.25	<0.001
	11	0.62	0.10	6.24	<0.001
	12	0.66	0.08	8.50	<0.001
	13	0.59	0.07	8.83	<0.001
	14	0.56	0.08	7.03	<0.001
	15	0.53	0.08	6.62	<0.001
	16	0.61	0.07	8.73	<0.001
	17	0.72	0.11	6.45	<0.001
	18	0.72	0.10	7.09	<0.001
	19	0.43	0.07	6.26	<0.001
Alterations in attention or consciousness	20	0.65	0.07	9.23	<0.001
	21	0.58	0.17	3.47	0.001
	22	0.82	0.07	11.80	<0.001
	23	0.73	0.06	11.32	<0.001
	24	0.65	0.07	9.23	<0.001
Alterations in self-perception	25	0.69	0.10	7.05	<0.001
	26	0.75	0.05	14.92	<0.001
	27	0.76	0.06	12.37	<0.001
	28	0.71	0.06	12.46	<0.001
	29	0.68	0.06	10.74	<0.001
	30	0.51	0.09	5.46	<0.001
Alterations in relationships with others	31	0.74	0.04	18.84	<0.001
	32	0.67	0.07	10.34	<0.001
	33	0.70	0.06	11.70	<0.001
	34	0.79	0.06	13.85	<0.001
	35	0.74	0.07	10.42	<0.001
Somatisation	36	0.65	0.08	8.06	<0.001
	37	0.73	0.06	11.51	<0.001
	38	0.81	0.08	9.87	<0.001
	39	0.75	0.09	8.65	<0.001
	40	0.73	0.10	7.70	<0.001
Alterations in systems of meaning	41	0.71	0.08	9.45	<0.001
	42	0.73	0.09	8.57	<0.001
	43	0.60	0.08	7.43	<0.001
	44	0.80	0.07	11.29	<0.001
	45	0.45	0.09	4.89	<0.001

**Table 3:** SIDES-SR scales’ means, standard deviations, and Cronbach’s alphas.

	SIDES-R (Pelcovitz et al., 1997 [1])	SIDES-SR Portuguese Version					
		Community (*N* = 814)			Clinical (*N* = 310)		
	α	*M*	*SD*	α	*M*	*SD*	α
I. Alt. regulation affect and impulses	0.90	0.18	0.33	0.90	0.67	0.61	0.77
II. Alt. attention or consciousness	0.76	0.18	0.50	0.68	0.67	0.82	0.62
III. Alterations in self-perception	0.77	0.07	0.21	0.78	0.37	0.52	0.69
IV. Alt. relationships with others	0.77	0.15	0.37	0.67	0.48	0.66	0.62
V. Somatisation	0.88	0.06	0.21	0.69	0.26	0.51	0.71
VI. Alt. systems of meaning	0.78	0.16	0.45	0.69	0.53	0.86	0.67

*Note.* Alt. regulation affect and impulses = alterations in regulation of affect and impulses; Alt. attention or consciousness = alterations in attention or consciousness; Alt. relationships with others = alterations in relationships with others; Alt. systems of meaning = alterations in systems of meaning. Acceptable alphas: >70 (>60 in exploratory studies) (Fachel and Camey, 2000) [43]. adapted with permission from Ref. [1]. 1997, Pelcovitz et al.

**Table 4:** Measurement structure invariance.

Model	Chi-Square	*df*	*p*	CFI	TLI	NFI	RMSEA	Chi-Square Diff	*df*	Diff Pr (>Chisq)
Model 1	1796.7	1774	0.348	0.999	0.999	0.910	0.005			
Model 2 ^1^	2087.7	1812	<0.001	0.985	0.984	0.895	0.018			
Difference								46.88	38	0.153
Model 3 ^2^	2051.4	1850	0.001	0.989	0.989	0.897	0.015			
Difference								−45.15	38	1

*Note.* ^1^ Model with equal loadings; ^2^ model with equal loadings and intercepts.

**Table 5:** SIDES-SR scales mean, Student’s *t*-test and effect sizes.

		Means	*t*	*p*	*d*
I. Alterations in regulation of affect and impulses	Community	0.18	−12.48	<0.001	−1.16
	Clinical	0.67			
II. Alterations in attention or consciousness	Community	0.18	−9.56	<0.001	−0.82
	Clinical	0.67			
III. Alterations in self-perception	Community	0.07	−9.40	<0.001	−0.91
	Clinical	0.37			
IV. Alterations in relationships with others	Community	0.15	−8.08	<0.001	−0.70
	Clinical	0.48			
V. Somatisation	Community	0.06	−6.23	<0.001	−0.61
	Clinical	0.25			
VI. Alterations in systems of meaning	Community	0.16	−6.92	<0.001	−0.63
	Clinical	0.52			

*Note.* The effect size Cohen’s *d* was considered small when: *d* ≤ 0.20, medium when: 0.20 < *d* ≤ 0.50, large when: 0.50 < *d* ≤ 1.0, and very large when *d* > 1.0 (Cohen, 1988 [39]).

## Data Availability

To obtain the dataset analysed for this study, please contact ruteoliveirapires@gmail.com or rpires@psicologia.ulisboa.pt.
